# A Method for Isolation Bacteriophage Particles-Free Genomic DNA, Exemplified by TP-84, Infecting Thermophilic *Geobacillus*

**DOI:** 10.3390/microorganisms10091782

**Published:** 2022-09-03

**Authors:** Ireneusz Sobolewski, Katarzyna Adamowicz, Anna Struck, Agnieszka Zylicz-Stachula, Piotr M. Skowron

**Affiliations:** Department of Molecular Biotechnology, Faculty of Chemistry, University of Gdansk, Wita Stwosza 63, 80-308 Gdansk, Poland

**Keywords:** TP-84, bacteriophage, thermophile, DNA purification, genomics, phage display

## Abstract

DNA purification methods are indispensable tools of molecular biology, used for many decades. Nevertheless, for certain specialized applications, the currently employed techniques are not sufficiently effective. While examining a number of the existing methods to purify the genomic DNA of the thermophilic bacteriophage TP-84, which infects *Geobacillus stearothermophilus* (*G. stearothermophilus*), we have found out that the obtained DNA is contaminated with trace amounts of infectious TP-84 particles. This was detrimental for the bacteriophage genetic manipulation purposes, as finding the recombinant TP-84 clones was essentially impossible due to the appearance of a high background of native bacteriophage plaques. Thus, we have developed a method, which enables the fast and efficient isolation of a bacteriophage genomic DNA from concentrated phage preparations, obtained using CsCl gradient ultracentrifugation, without the need to remove concentrated CsCl solutions. The method employs silica columns and mini-scale isolation of microgram amounts of high quality DNA. It is universal—the silica mini-columns from various manufacturers can be used to conduct the procedure. The purified DNA, free from infectious bacteriophage particles, is ready for further manipulations. This is particularly important for such thermophilic bacteriophages that may partially survive standard isolation procedures and contaminate the final DNA product.

## 1. Introduction

Bacteriophages—the viruses that infect bacteria, are highly useful both for basic research and biotechnological application. These relatively simple biological models and virus-bacterial host interactions were originally used to develop molecular genetics, starting from the early 20th century. Their practical applications in biotechnology include phage therapy, phage display systems, vaccine scaffolds, nano-vehicles for gene delivery, food biopreservation, bacterial biosensing devices, nanomaterials and others [1]. Although the thermophilic bacteriophages are rare objects of research, they are interesting because of their often atypical molecular biology and potential applications in molecular biotechnology [2]. Their resistance to a wide temperature range and harsh chemical environment makes them useful scientific research models in areas not accessible for standard model microorganisms. One example of such a thermophilic bacteriophage is TP-84, which was isolated in 1952 from greenhouse soil using *G*. *stearothermophilus* as a host [3]. Since then, TP-84 has become extensively characterized, with a contribution from our laboratory work to a deeper understanding of the genome organization and proteomics [4,5,6,7]. This allowed us to construct a new phage-display system with a potential use in protein engineering, drug discovery and diagnostics (to be published elsewhere). Such a new thermostable phage display system could be used in the pharmaceutical industry to develop and produce new vaccines, biological drugs, protect the environment (plant protection products; elimination of pollution) and in other industrial applications. 

To construct a phage-display system or conduct other genetic manipulations on a bacteriophage genome, the isolation of high-quality genomic DNA from the TP-84 bacteriophage is an obligatory requirement. Thus, before the genomic DNA of TP-84 is extracted, the pure particles of this bacteriophage need to be first obtained. There are several methods used to purify bacteriophages particles [8,9,10]. The most common one is polyethylene glycol (PEG) precipitation [11] and CsCl gradient centrifugation [12,13,14,15]. After centrifugation in a CsCl gradient, the DNA isolation requires a downstream removal of concentrated CsCl by dialysis [16,17], as it inhibits the activities of enzymes used in the subsequent stages of isolation as well as bacteriophage DNA cloning and analysis. Furthermore, isolation of a bacteriophage DNA from CsCl-purified particles typically employs organic reagents harmful to health and the environment (phenol; chloroform) [9,13,15,18]. According to our knowledge, there is actually only one commercially available kit dedicated to the isolation of bacteriophage DNA from phage cultures (NorgenBiotek, Thorold, ON, Canada). However, the starting material for this procedure is the clarified culture supernatant, obtained as a result of bacteriophage cell lysis, that has been separated from bacterial debris by centrifugation. Thus, minute amounts of DNA are obtainable from such diluted sample and the complete removal of infectious bacteriophage particles was out of our hands. In the present project, we developed a novel method, which enables a fast and efficient isolation of bacteriophage genomic DNA from concentrated CsCl preparations without the time-consuming removal of CsCl. The protocol, though originally developed for TP-84, is universal and can be used for other bacteriophages.

## 2. Materials and Methods

### 2.1. Bacterial Strains, Media and Reagent

*G. stearothermophilus* 10 (StrR)—streptomycin-resistant mutant was constructed and used as a host for bacteriophage TP-84 and grown in the TYM medium supplemented with streptomycin (Sigma-Aldrich, St. Louis, MO, USA) at 50 µg/mL. Components of TYM medium contained: Peptone K (pancreatic casein hydrolysate) (BTL, Łódź, Poland), yeast extract (BTL), MgCl_2_ (Sigma-Aldrich, St. Louis, MO, USA), CaCl_2_xH_2_O (Merck KGaA Darmstadt, Germany) and fructose (Bioshop Burlington, Canada). For precipitation of the bacteriophage particles, PEG 8000 (Bioshop) was used. TP-84 particles were suspended in TMC buffer: 10 mM Tris-HCl (Sigma-Aldrich), 10 mM MgSO_4_ (Stanlab, Lublin, Poland), 5 mM CaCl_2_ and pH 7.0. CsCl used for density gradient was from (Sigma-Aldrich). The enzymes used in the DNA isolation procedure were RNase A (GeneON GmbH, Ludwigshafen, Germany), DNase I (F. Hoffmann-La Roche Ltd., Basel, Switzerland) and proteinase K (AppliChem Inc., Darmstadt, Germany). The DNase I stocks used here were prepared in a buffer 50% glycerol, with 10 mM Tris-HCl pH 7.5, 50 mM CaCl_2_, 10 mM MgCl_2_ at a concentration of 10 mg/mL and stored in 0.05 mL aliquots at −20 °C. The DNase I 10× buffer contained 100 mM Tris HCl pH 7.5, 25 mM MgCl_2_ and 5 mM CaCl_2_. DNA purification kit components were from undisclosed suppliers (S1–S4) to avoid any bias and incidental advertising. The BglII restriction endonuclease was from New England Biolabs (Ipswich, MA, USA). The DNA standards were from ThermoFisher Scientific Baltics UAB (Vilnus, Lithuania). All the other chemicals were from Sigma-Aldrich. Spin-columns SCUN1 and SCUN2 were prepared in our laboratory using various commercial plastic spin columns, which were fitted with 4 layers of glass filter membranes (Munktell MGB). The PCR reaction and restriction endonuclease digestions were designed using the SnapGene software version 5.1.5 (http://www.snapgene.com) (accessed on 29 May 2022).

### 2.2. Bacteriophage Propagation and Purification

#### 2.2.1. TP-84 Cultivation

The bacteriophage TP-84 was cultivated by infecting the bacterial host strain *G*. *stearothermophilus* 10 (StrR), using 1 L of rich medium supplemented with calcium ions, magnesium ions and fructose. The liquid TYM medium contained (per 1 L): Peptone K 20 g, yeast extract 4 g, MgCl_2_ 10 mM, CaCl_2_ 5 mM and fructose 0.5%. In the first step (day 1), the fresh host culture was prepared. A total of 20 mL of TYM medium was inoculated with *G. stearothermophilus* 10 (StrR) and shaken overnight at 55 °C. Then (day 2), 1 L of TYM medium was inoculated with the overnight culture of the host to an OD_600_ of 0.1. Culturing was conducted at 55 °C with vigorous aeration by shaking in 5 L flasks at 220 rpm. The infection was performed when OD_600_ reached 0.2 ± 0.3, then an additional fructose portion was added to 0.5% and the culture was grown for an additional 30 minutes (min) until an OD_600_ of 0.4 ± 0.5 and was infected with TP-84 at M.O.I. of 0.01. The culture was left for 10 min at 55 °C without shaking, to allow efficient adsorption of the bacteriophage. Shaking was then started and continued until an OD_600_ value of 0.1 was reached; the cultivation was terminated by adding 3 mL of chloroform and left for 20 min at 55 °C with low speed shaking. The bacteriophage quantification was conducted using the soft-agar overlay technique [4]. The overlayer contained 3 mL of 0.65% agar in TYM broth, 0.3 mL of a cell suspension of *G. stearothermophilus* 10 and 5 µL of the purified phage DNA. The base layer contained about 30 mL of 2% agar in the same broth (TYM agar). The plates were incubated overnight at 55 °C prior to the counting of plaques.

#### 2.2.2. TP-84 Particles Purification

The bacteriophage particles were purified using selective precipitation, followed by CsCl ultracentrifugation [12]. The bacterial lysate was centrifuged (10,000× *g*, 10 °C, 20 min). NaCl and PEG 8000 were added to the supernatant to a concentration of 0.5 M and 5%, respectively, and stirred in a refrigerator overnight on a magnetic stirrer. The mixture was centrifuged (9000× *g*, 4 °C, 40 min) and the white pellet, containing the bacteriophage, was resuspended in 5 mL of TMC buffer. The suspension was extracted three times with chloroform after centrifugation (4000× *g*, 4 °C, 10 min). The upper aqueous layer was collected and applied to a CsCl gradient. The gradient was prepared by the layering of CsCl solutions with densities of: 1.7 g/cm^3^, 1.5 g/cm^3^ and 1.3 g/cm^3^, respectively. The separation was performed by centrifuging at 80,000× *g* for 2.5 h at 10 °C in the Rotor Sur Spin 630/17 Thermo Scientific (17 mL). The band, containing TP-84, was collected using a needle and syringe (Figure S1).

#### 2.2.3. *G. stearothermophilus* Selection

*G. stearothermophilus* 10 mutant for simplified, contamination-free TP-84 cultivation was selected using serial passaging on solid TYM media using increasing streptomycin concentrations: 5 µg/mL, 10 µg/mL, 20 µg/mL, 50 µg/mL. The samples of 300 μL of overnight *G. stearothermophilus* 10 culture (app. 2 × 10^8^ cells) were plated on TYM plates. Upon acquiring resistance of *G. stearothermophilus* at 50 µg/mL streptomycin concentration, serial passagings were conducted to ensure the mutant stability.

## 3. Results and Discussion

### 3.1. Method Development and Validation

**Figure 1:**
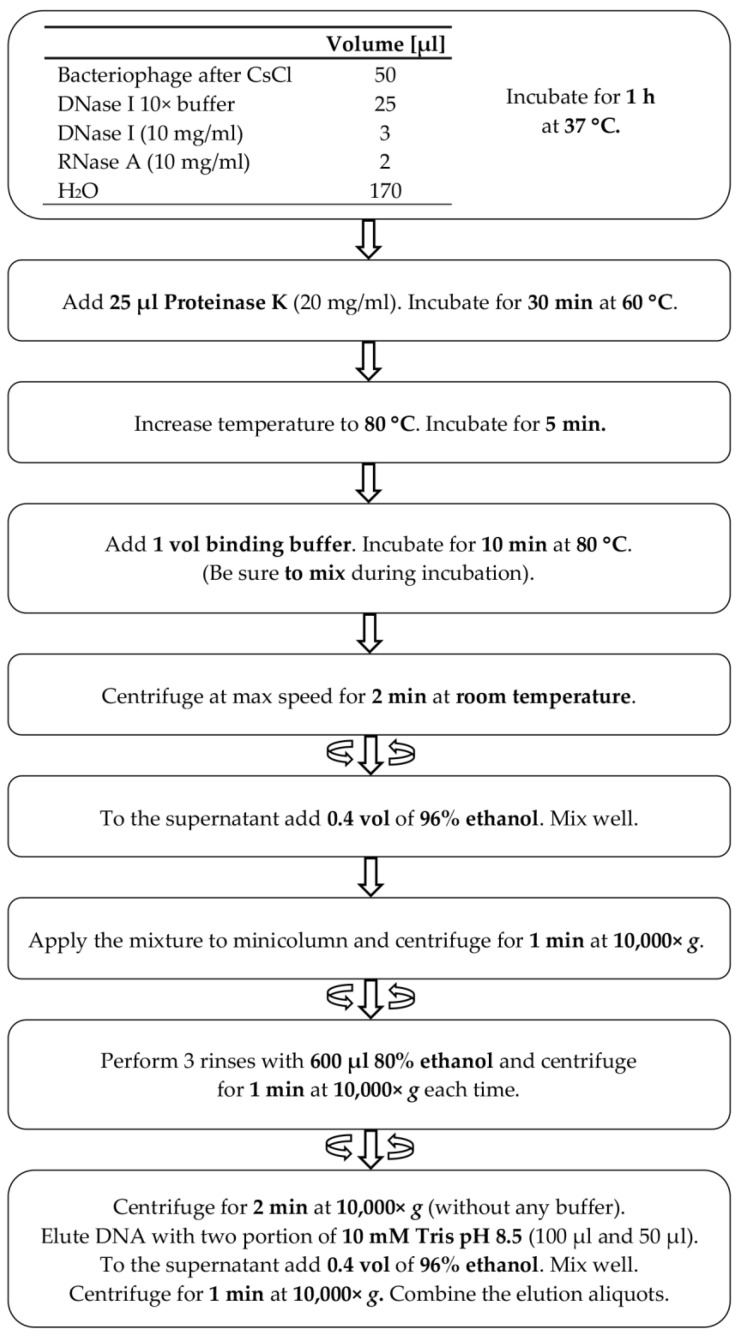
Scheme illustrating the method for isolation of bacteriophage genomic DNA, free of bacteriophage particles.

**Figure 2:**
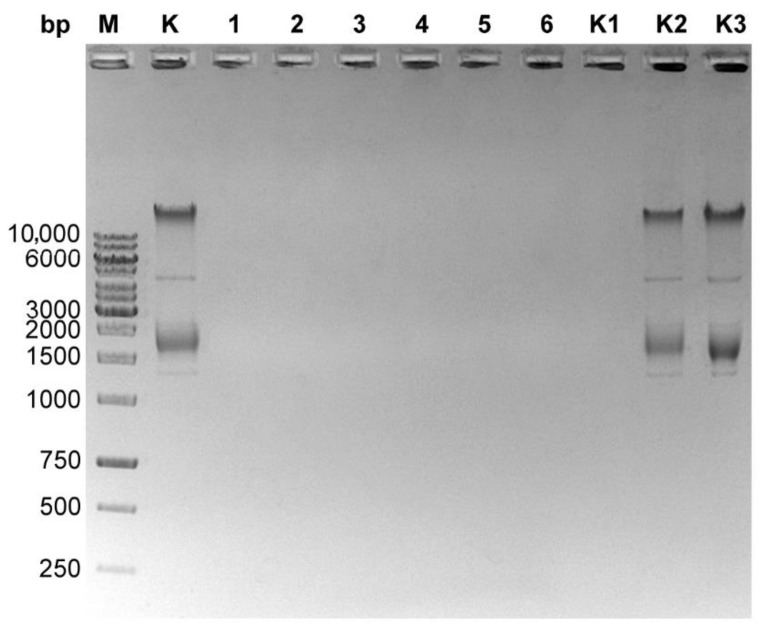
Digestion of *E. coli* genomic DNA with DNase I (new batch, glycerol stock) in the presence of various CsCl concentrations. A total of 0.4 µg of *E. coli* genomic DNA was digested with 4 µg DNase I at 37 °C for 30 min and inactivated at 80 °C for 15 min. Lane M, GeneRuler 1 kb DNA Ladder; lane K, undigested *E. coli* DNA (0.4 µg); lanes 1–6, samples with CsCl diluted 1:10, 1:9, 1:8, 1:7, 1:6 and 1:5; lane K1, sample without CsCl; lane K2, sample without DNase I, CsCl diluted 1:20; lane K3, sample without DNase I, CsCl diluted 1:5.

**Figure 3:**
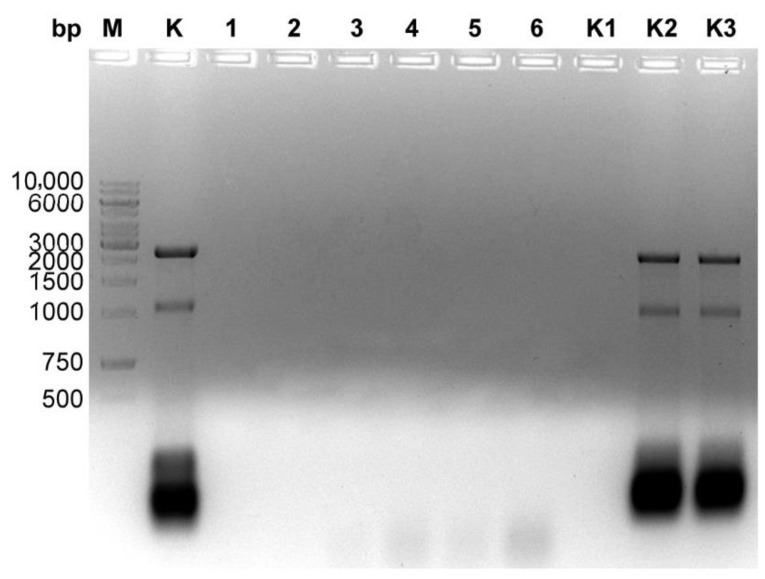
Digestion of *E. coli* RNA with RNase A in different concentration of CsCl. A total of 7 µg of *E. coli* RNA was digested with 3.5 µg RNase A at 37 °C for 30 min. RNase-digested RNA was purified using a RNA Clean-Up kit. A total of 1.2% agarose gel electrophoresis: Lane M, GeneRuler 1 kb DNA Ladder; lane K, undigested *E. coli* RNA (7 µg); lanes 1–6, samples with CsCl diluted 1:10, 1:9, 1:8, 1:7, 1:6 and 1:5; lane K1, a sample without CsCl; lane K2, sample without RNase A and CsCl; lane K3, sample without RNase A and CsCl diluted 1:5.

**Figure 4:**
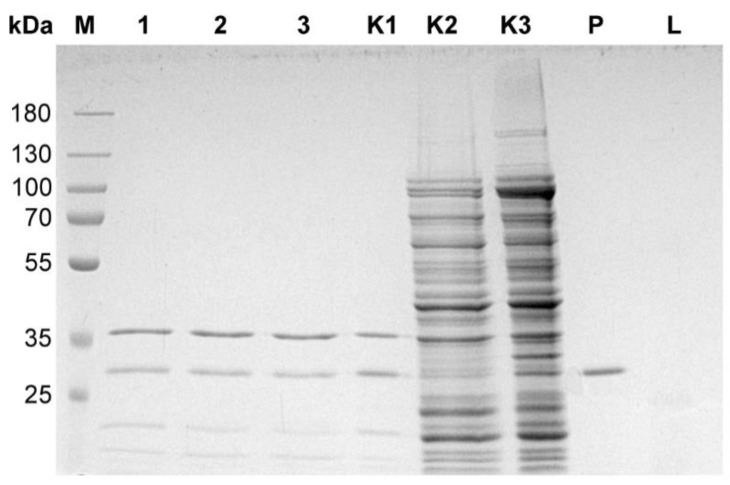
Effect of the presence of various concentrations of CsCl on the activity of proteinase K. Lysate *E. coli* pPR B5 (0.25 mL culture, OD_600_ = 3.6) was digested with 48 µg of proteinase K in various concentrations of CsCl at 55 °C for 30 min. SDS-PAGE/glycine analysis, 12% gel: Lane M, protein marker (PageRuler, Prestained Protein Ladder, 10 to 180 kDa); lanes 1–3 samples with CsCl diluted 1:10, 1:7, 1:5; lane K1, sample without CsCl; lane K2, sample without proteinase K, CsCl diluted 1:5; lane K3, sample without CsCl and proteinase K; lane P, proteinase K (80 µg); lane L, lysozyme (50 µg).

The general outline of the method (Figure 1) is a modified variant of commonly used protocols of genomic DNA isolation. Initially, particles of a bacteriophage are disintegrated through an enzymatic and chemical lysis process with an appropriate lysis buffer. Ethanol is added to the lysate, containing the released bacteriophage genomic DNA, and the solution is loaded onto a silica spin-column in the mini format for Eppendorf tubes. The isolation procedure involves the use of three enzymes: DNase I, RNase A and proteinase K. In the first step, DNase I and RNase A remove the residual DNA and RNA from the bacterial host cells, prior to releasing a bacteriophage DNA from the capsids, then proteinase K inactivates DNase I and RNase A, weakens the bacteriophage capsid and, after the lysis of phage particles, removes proteins from the DNA-protein complex. Typically, the range of 2.4–5.6 M (1.3 g/cm^3^−1.7 g/cm^3^) CsCl is used in gradient centrifugation [13,14,15]. Such a high concentration of salt virtually inhibits the activities of enzymes [19,20]. The most sensitive of the three enzymes used is DNase I (Figure S2). To increase the DNase I stability in the reaction, it was concluded that it would be safe to use a five-fold dilution of CsCl solution. According to our results, a 1:4 dilution (5×) of the 5.6 M CsCl solution (1.12 M finally) was sufficient to unlock the proper enzymatic functioning of DNase I (Figure 2) as well as RNase A (Figure 3) and proteinase K (Figure 4). In this way, the need for buffer exchange through dialysis was omitted, which is highly advantageous in the development of a rapid bacteriophage DNA preparation. Such an approach saves time and reagents, enhancing the economy and usability of the method. Furthermore, as the initial CsCl pre-purification removes the bulk of host material, there is no need for DNA precipitation or extraction with phenol.

**Figure 5:**
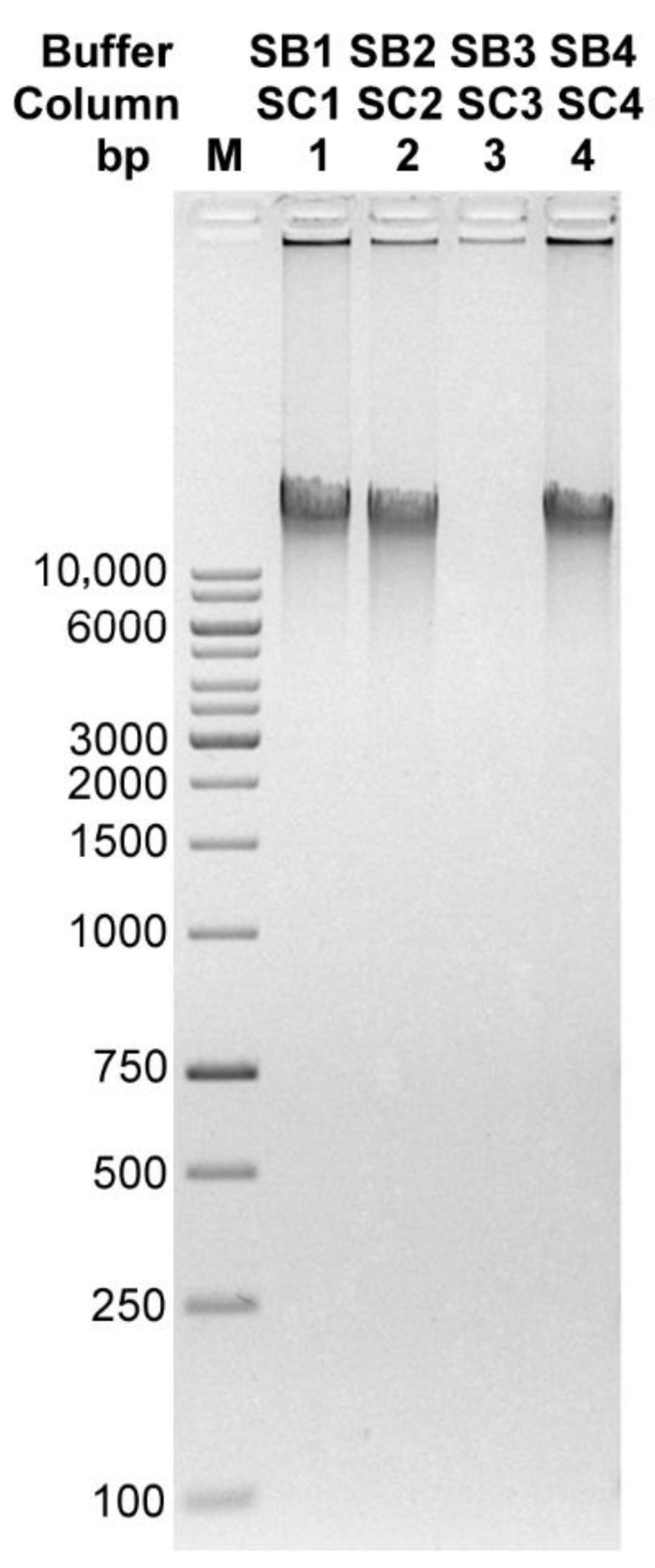
DNA of bacteriophage TP-84 extracted with four different commercially available lysis buffers together with dedicated spin-columns. Samples were electrophoresed on 1% agarose/TBE gel. Lane M1, GeneRuler 1 kb DNA Ladder; lane 1, Supplier S1; lane 2, Supplier S2; lane 3, Supplier S3; lane 4, Supplier S4.

**Figure 6:**
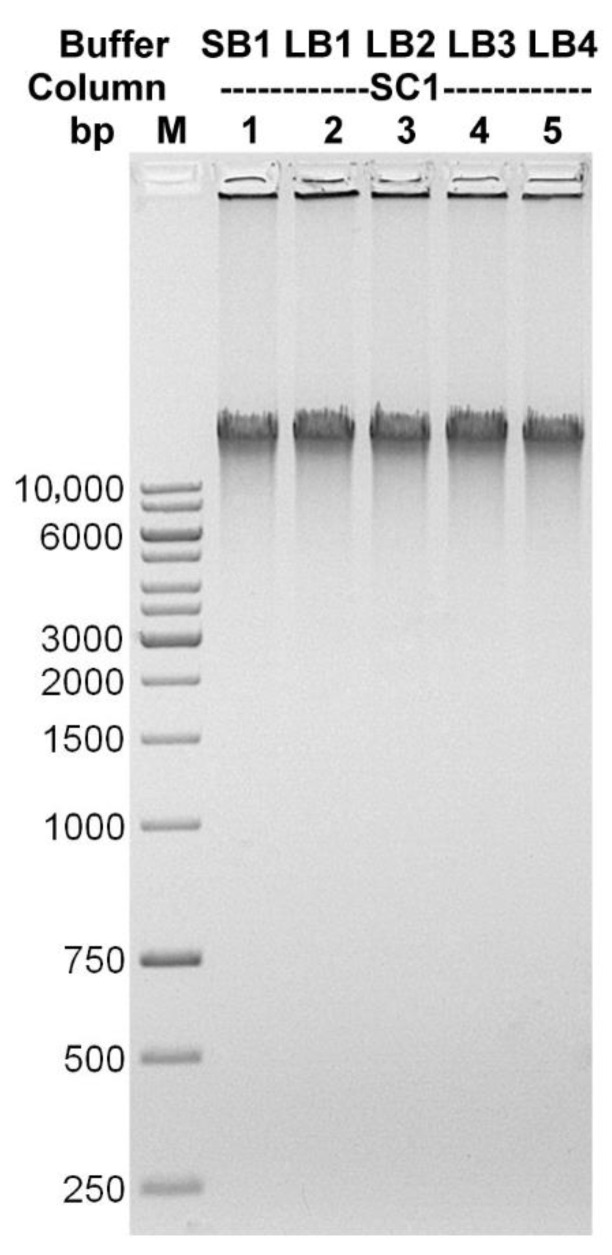
Bacteriophage TP-84 DNA isolated using spin-columns obtained from the Supplier S1 and here developed lysis buffers. Samples were electrophoresed on 1% agarose/TBE gel. Lane M, GeneRuler 1 kb DNA Ladder; lane 1, Supplier S1 buffer; lane 2, lysis buffer LB1; lane 3, lysis buffer LB2; lane 4, lysis buffer LB3; lane 5, lysis buffer LB4.

**Figure 7:**
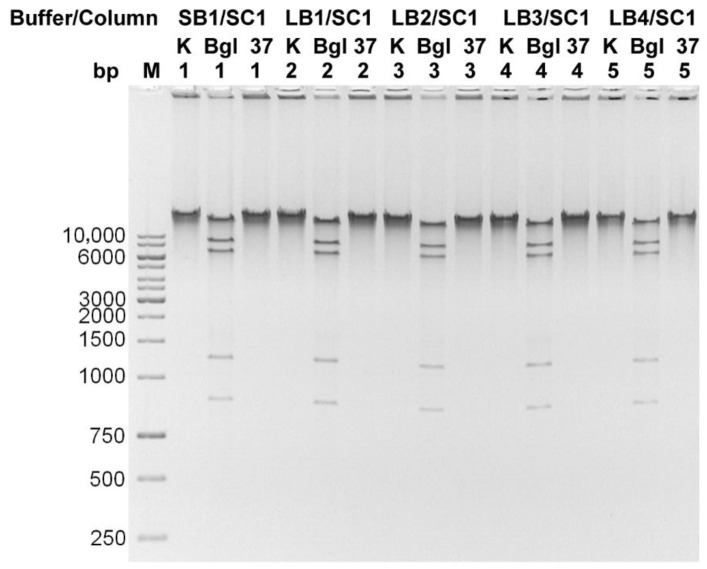
Bacteriophage TP-84 DNA isolated using spin-columns obtained from the Supplier S1 and developed lysis buffers. The quality of the obtained DNA was tested by cleavage with BglII restriction endonuclease at 37 °C for 20 min (Bgl) and control incubation at 37 °C for 1 h (37) in the BglII digestion buffer. Samples were electrophoresed in 1% agarose/TBE gel. Lane K—undigested DNA; lane M, GeneRuler 1 kb DNA Ladder; lanes 1, Supplier S1 buffer; lanes 2, lysis buffer LB1; lanes 3, lysis buffer LB2; lanes 4, lysis buffer LB3; lanes 5, lysis buffer LB4.

After optimizing the conditions of the enzymatic cocktail operation, the performance of four commercially available lysis buffers, together with dedicated mini-columns, were compared. All the following isolations were performed on a bacteriophage sample with a titer of 1 × 10^11^ particles/mL after CsCl gradient (25 µL for one isolation). The TP-84-containing gradient band was collected in the range between 1.5 g/cm^3^−1.7 g/cm^3^ CsCl. The maximum possible concentration of CsCl in the preparation was 5.6 M. After incubation with the enzymes, 1 volume of each of the lysis buffers was added to the series of diluted CsCl solutions with bacteriophage particles and incubated for 10 min at 80 °C. The mixtures were then centrifuged for 2 min at maximum speed. The supernatants were transferred to new Eppendorf tubes, where 0.4 volumes of 96% ethanol were added and the mixture was applied to manufacturer-provided spin columns. After being washed three times with ethanol, the DNA was eluted in two portions with 10 mM Tris-HCl pH 8.5, 2 × 75 µL. Under these conditions, the best effect was obtained when using the Supplier’s S1 and S4 buffers/columns combinations (Figure 5). The precise concentrations of DNA preparations are given in Table 1. It is worth emphasizing that the DNA purification kits used here were not dedicated to the purification of bacteriophage DNA and not from such crude bacteriophage DNA solutions. Instead, according to our protocol, the genomic DNA is purified from concentrated CsCl solutions. The components of the kits were used only as starting elements to develop and optimize our new method. Our lysis buffers were formulated during optimization steps. In order to increase the isolation efficiency, several binding buffers were evaluated, varying in detergent concentrations. These were used in conjunction with the Supplier S1 columns (SC1) and compared to the Supplier S1 buffer (SB1) (SB1/SC1 best performing in the previous test) (Figure 6). The precise concentrations of DNA preparations are given in Table 2. All lysis buffers contained: (i) EDTA to weaken the integrity of the bacteriophage capsid, which requires calcium ions for stability, (ii) guanidine hydrochloride to bind DNA to silica membranes and (iii) detergents to lyse the bacteriophage particles and purify DNA from proteins and other compounds. The buffer compositions are presented in Table S1. Other isolation conditions were the same as shown above. The quality of the obtained DNA was checked by digesting with BglII restriction endonuclease, which was selected due to its clear digestion patter on TP-84 genomic DNA (Figure 7). All of the new buffers work well with SC1 spin-columns and give good quality DNA. Buffers LB1 and LB2 were selected for further tests. In the next step, the isolation efficiency and compatibility of the new buffers and six different silica spin-columns from various manufacturers were evaluated. Purifications were performed according to the protocol described above. The DNA was separated in 1% agarose gel stained with ethidium bromide. The results are shown in Figure S3 and Table S2.

All the tested spin-columns contained silica/fiber glass membranes and were miniprep scale. TP-84 genomic DNA was obtained using each buffer/spin-column combination. The cross tests of various manufacturers’ spin columns used here did not exhibit significant differences, thus confirming the expected universality of the generally used mini format spin-column/silica/glass fiber membranes. Therefore, essentially, any silica-based spin-column seems to be suitable for the method developed here, with minor differences in DNA yields. The LB1 buffer is the least compatible with the SC3 column (the smallest amount of DNA, 3.79 µg). The LB2 buffer gives a better result, for the weakest combination with an SC3 column being 1.28 µg more DNA than the LB1 buffer. The overall results for both buffers are very similar and it is difficult to determine which is better. There is a 3.45 µg difference between the best and the weakest for LB1 buffer (SC3 and SCUN1) and 2.78 µg for LB2 (SC2 and SCUN2). For the best performing spin-columns—SCUN1 and SCUN2, this difference between the buffers was insignificant. At this stage, the LB2 buffer seems to be more universal. Thus, it is recommended to conduct a test isolation, prior to the use of a new silica spin-column, as the membranes used may vary depending on the manufacturer. Further isolations of bacteriophage TP-84 DNA, according to our protocol, will be carried out with the use of the LB2 buffer.

### 3.2. Genomic DNA Contamination with Bacteriophage Particles Problem

The absence/presence of viable TP-84 bacteriophages in the purified genomic DNA preparations was determined by the soft-agar overlay technique [4], on TYM-based-double-layered agar plates inoculated with *G. stearothermophilus* 10 (StrR) and then evaluated for the presence or absence of plaque formation. In some cases, live TP-84 bacteriophages were observed, which could be inactivated by heating the preparation in 95 °C for 15 min (Figure S4). However, this treatment was decreasing the DNA quality both in terms of visual assessment on an electrophoretic gel as well as in the restriction or PCR reactions (not shown).

**Figure 8:**
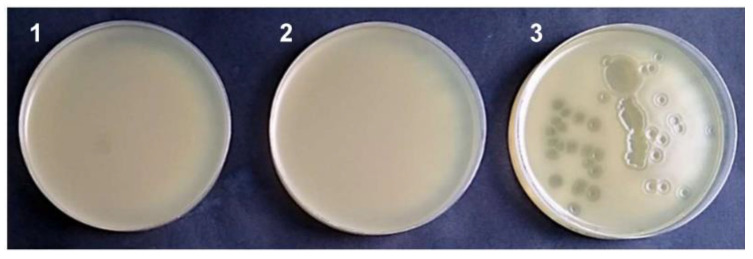
The effect of mixing of the reaction mixture at the stage of lysis and incubation in 80 °C on the viability of contaminating TP-84 bacteriophage. The *G. stearothermophilus* 10 (StrR) was mixed with 5 µL of the purified TP-84 genomic DNA. The mixture was embedded in 0.65% agar TYM broth and incubated on solid agar plates at 55 °C: 1, with host bacterium only; 2, TP-84 DNA isolated with thorough mixing, CsCl diluted 1:5; 3, TP-84 DNA isolated without mixing and CsCl diluted 1:5.

After a thorough analysis of each step of the procedure developed, the cause of contamination of the preparations turned out to be the insufficient mixing of the initial lysis mixture and its incubation in 80 °C (Figure 8). We conclude that very few bacteriophage particles can escape lysis, which is especially true in the case of thermophilic ones if they are trapped in the micro pores of the spin-columns or the cavities between the cap and the top of a tube or mini-column. Thus, this seemingly trivial manipulation is also a critical factor in the bacteriophage genomic DNA purification, especially if it is intended for genetic manipulations, such as those performed using the phage display technique. 

### 3.3. Isolation of a Bacteriophage Particles-Free Bacteriophage Genomic DNA. Final Protocol

**1.** Purify bacteriophage particles using standard PEG/NaCl precipitation, followed by CsCl gradient ultracentrifugation; collect the opaque band, containing a bacteriophage, and keep the amount of surrounding CsCl solution to minimum.

**2.** Conduct lysis of 50 µL in a bacteriophage preparation with preferably a high titer of approximately 4 × 10^11^ or more particles/mL per one mini-column in 250 µL reaction, using a 2.0 mL Eppendorf tube according to Table 3. As much as 100 µL of a bacteriophage preparation can be used. In such a case, the reaction should be brought to 500 µL volume with water. After one hour of incubation at 37 °C, increase the temperature to 60 °C and add 25 µL of proteinase K (20 mg/mL). Incubate at 60 °C for 30 min.

**3.** Inactivate the enzymes by heating the sample at 80 °C for 5 min.

**4.** Complete a bacteriophage lysis by the addition of one volume (275 µL or 550 µL if 100 µL of a bacteriophage preparation was used) of binding buffer (2.5 M guanidine hydrochloride, 25 mM EDTA, 0.5% Tween 20, 1% Triton X-100), mix thoroughly by inverting the tube several times and heat the sample for 10 min at 80 °C. During the incubation, mix the sample again several times by inverting the tube. After every mixing, centrifuge the sample at 5000× *g* for 15 s. Following this mixing scheme at this stage, it is essential to remove any surviving bacteriophage particles. Upon lysis completion, centrifuge at 16,000× *g* for 2 min and transfer the supernatant to a new Eppendorf tube.

**5.** Add 0.4 volumes of ethanol (96%) and mix thoroughly, then transfer the mixture to a mini-column and centrifuge at 10,000× *g* for 1 min. Remove and discard the filtrate, add 600 µL washing solution (80% ethanol) to mini-column and centrifuge at 10,000× *g* for 1 min. Discard the filtrate and return the mini-column to the tube. Repeat washing twice. 

**6.** Dry the membrane by centrifuging the mini-column for 3 min at 10,000× *g*.

**7.** Place the mini-column into a new Eppendorf tube and elute DNA with 100 µL of elution buffer (10 mM Tris-HCl pH 8.5). After 2 min incubation, centrifuge the sample for 1 min at 10,000× *g* and repeat the elution with 50 µL of elution buffer. The yield will depend on a bacteriophage type, titer of initial CsCl preparation and adhering to the protocol. For TP-84, we were repeatedly obtaining large amounts of approximately 32 µg of DNA from a single tube.

## 4. Conclusions

1. A novel method for high purity genomic DNA isolation in a mini spin-format was developed and dedicated to concentrated CsCl preparations, as opposed to isolations performed directly for bacteriophage culture lysates used thus far.
2. The method enables the fast and efficient purification of bacteriophage genomic DNA from concentrated bacteriophage preparations without removing concentrated CsCl solutions.
3. The purified DNA is ready for further applications and analyzes without the need for precipitation or extraction with phenol.
4. The protocol eliminates false ‘positives’ background during recombinant bacteriophages construction, such as when conducting phage display experiments.
5. The preparations are free from alive bacteriophage particles, which is especially important in the case of highly resistant thermophilic bacteriophages that can survive the isolation procedures and contaminate the final DNA, ultimately leading to a contamination of laboratory surfaces and future host cultures.

## Figures and Tables

**Table 1:** DNA concentration and purity determined by spectrophotometric analysis, using commercially available DNA purification kits.

Buffer/Column	DNA Concentration [ng/µL]	260/280	260/230	Amount of DNA Obtained [µg]
SB1/SC1	33.67	1.97	1.79	5.05
SB2/SC2	26.41	1.77	2.36	3.96
SB3/SC3	3.22	1.98	1.07	0.48
SB4/SC4	33.3	1.76	1.99	4.99

**Table 2:** DNA concentration and purity determined by spectrophotometric analysis, obtained using here developed lysis buffers.

Lysis Buffer	DNA Concentration [ng/µL]	260/280	260/230	Amount of DNA Obtained [µg]
SB1	38.88	1.9	1.53	5.83
LB1	37.85	1.9	1.75	5.68
LB2	36.68	1.89	1.7	5.5
LB3	35.81	1.89	1.74	5.37
LB4	30.51	1.96	1.84	4.58

**Table 3:** Components of the bacteriophage reaction mixture.

	Volume [µL]
TP-84 after CsCl gradient	50
DNase I 10× buffer	25
DNase I (10 mg/mL)	3
RNase A (10 mg/mL)	2
H_2_O	170

## Data Availability

The raw data concerning this study are available on request from the corresponding author.

## Supplementary Materials

The following supporting information can be downloaded at: https://www.mdpi.com/article/10.3390/microorganisms10091782/s1, Figure S1: Bacteriophage TP-84 band after centrifugation in CsCl gradient; Figure S2: The effect of old batch, water stock DNase I on digestion of *Escherichia coli* (*E. coli*) genomic DNA; Figure S3: Bacteriophage TP-84 DNA isolated using six commercially available silica spin-columns and proposed lysis buffers; Figure S4: The effect of temperature on the survival of TP-84 bacteriophage, contaminating purified DNA; Table S1: Compositions of lysis buffers, developed in this work; Table S2: DNA concentration and purity determined by spectrophotometric analysis.
