# Supplementary material for: A Method for Isolation Bacteriophage Particles-Free Genomic DNA, Exemplified by TP-84, Infecting Thermophilic Geobacillus
Source: Microorganisms. 2022 Sep 3;10(9):1782. doi: 10.3390/microorganisms10091782 (PMC9502220; doi:10.3390/microorganisms10091782)
Supplement: Supplementary file 1 [file microorganisms-10-01782-s001.zip › Figure S2.pdf]

The effect of old batch, water stock DNase I on digestion of *Escherichia coli* (*E. coli*) genomic DNA

It is known that DNaseI gradually loses its enzymatic activity during storage in a solution, even as frozen preparations. We have also confirmed, that our older DNase I batch (prepared in water and stored in aliquots upon rapid freezing) was less effective (Fig. S2) than a new batch (Fig. 2). Thus, we recommend using fresh preparations (when using water stocks) or glycerol stocks described in Materials and Methods, which are more stable.

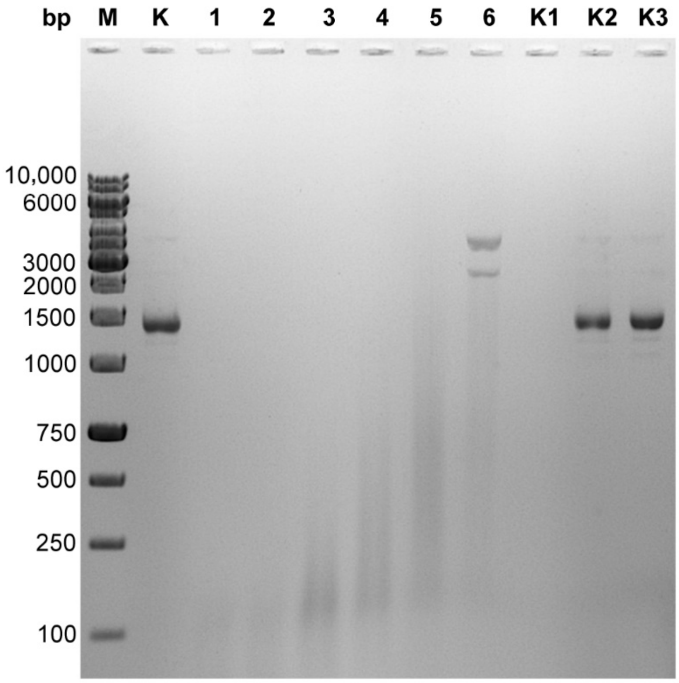

Figure S2. Digestion of *Escherichia coli* (*E. coli*) genomic DNA with DNase I (old batch, water stock) in the presence of various CsCl concentrations. 0.4 µg of *E. coli* genomic DNA was digested with 4 µg of DNase I at 37°C for 30 min and inactivated at 80°C for 15 min. Lane M, GeneRuler 1 kb DNA Ladder; lane K, undigested *E. coli* DNA (0.4 µg); lanes 1-6, samples with CsCl diluted 1 : 10, 1 : 9, 1 : 8, 1 : 7, 1 : 6 and 1 : 5; lane K1, sample without CsCl; lane K2, sample without DNase I, CsCl diluted 1 : 20; lane K3, sample without DNase I, CsCl diluted 1 : 5.
