# Supplementary material for: A Method for Isolation Bacteriophage Particles-Free Genomic DNA, Exemplified by TP-84, Infecting Thermophilic Geobacillus
Source: Microorganisms. 2022 Sep 3;10(9):1782. doi: 10.3390/microorganisms10091782 (PMC9502220; doi:10.3390/microorganisms10091782)
Supplement: Supplementary file 1 [file microorganisms-10-01782-s001.zip › Figure S3.pdf]

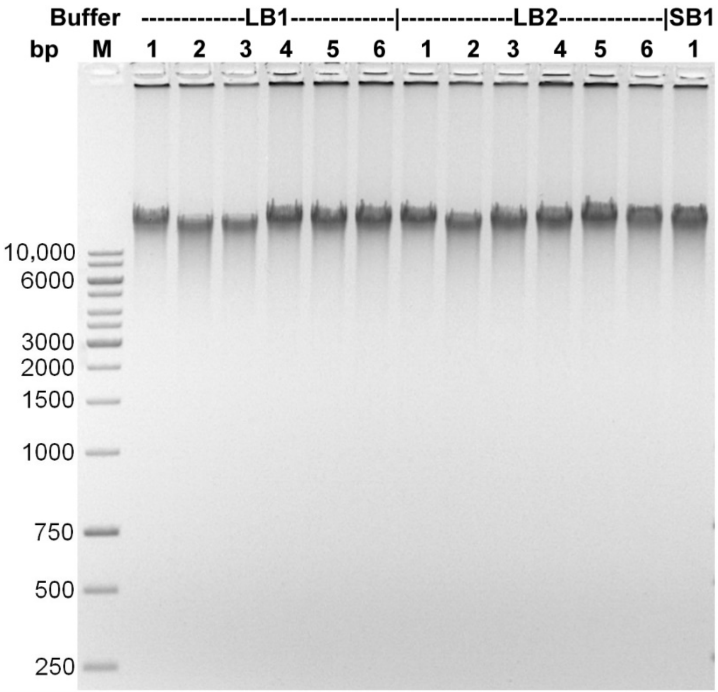


**Figure S3.** Bacteriophage TP-84 DNA isolated using six commercially available silica spin-columns and proposed lysis buffers: LB1, LB2 and Supplier 1 buffer as a control (SB1). Samples were electrophoresed in 1% agarose/TBE gel. Lane M, GeneRuler 1 kb DNA Ladder; lane 1, Supplier S1 spin-column; lane 2, Supplier S2; lane 3, Supplier S2; lane 4, Supplier unknown 1; lane 5, Supplier unknown 2; lane 6, Supplier 4 .
