# Supplementary material for: A Method for Isolation Bacteriophage Particles-Free Genomic DNA, Exemplified by TP-84, Infecting Thermophilic Geobacillus
Source: Microorganisms. 2022 Sep 3;10(9):1782. doi: 10.3390/microorganisms10091782 (PMC9502220; doi:10.3390/microorganisms10091782)
Supplement: Supplementary file 1 [file microorganisms-10-01782-s001.zip › Figure S4.pdf]

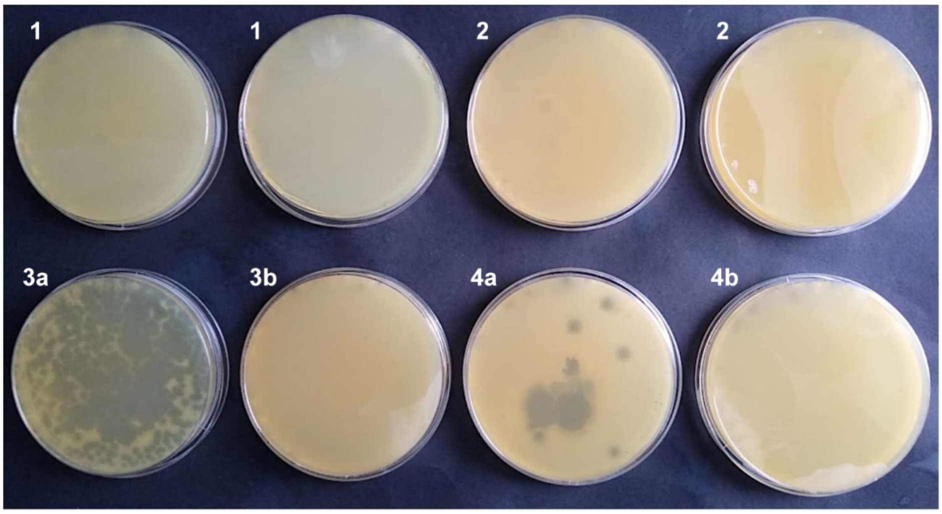

Figure S4. The effect of temperature on the survival of TP-84 bacteriophage, contaminating purified DNA. The host *G. stearothermophilus* 10 (StrR) was infected withTP-84 present in the purified genomic DNA. The mixture was embedded in 0.65% agar TYM broth and incubated (controls in duplicates) on sold agar plates at 55ºC: 1, only the host bacterium; 2, elution buffer; 3a, the isolated genomic DNA TP-84 – sample 1; 3b, DNA TP-84 – sample 1, incubated at 95ºC for 15 min; 4a, DNA TP-84 – sample 2; 4b, DNA TP-84 – sample 2, incubated in 95ºC for 15 min.
