# Supplementary material for: A Method for Isolation Bacteriophage Particles-Free Genomic DNA, Exemplified by TP-84, Infecting Thermophilic Geobacillus
Source: Microorganisms. 2022 Sep 3;10(9):1782. doi: 10.3390/microorganisms10091782 (PMC9502220; doi:10.3390/microorganisms10091782)
Supplement: Supplementary file 1 [file microorganisms-10-01782-s001.zip › Table S1.pdf]

Table S1. Compositions of lysis buffers, developed in this work.

**Lysis Buffer**	**LB1**	**LB2**	**LB3**	**LB4**
GuHCl	2.5 M	2.5 M	5 M	5 M
EDTA	25 mM	25 mM	50 mM	50 mM
Tween-20	1%	0.5%	1%	1%
Triton X-100	0.5%	1%	0.5%	-
