# Supplementary material for: A Method for Isolation Bacteriophage Particles-Free Genomic DNA, Exemplified by TP-84, Infecting Thermophilic Geobacillus
Source: Microorganisms. 2022 Sep 3;10(9):1782. doi: 10.3390/microorganisms10091782 (PMC9502220; doi:10.3390/microorganisms10091782)
Supplement: Supplementary file 1 [file microorganisms-10-01782-s001.zip › Table S2.pdf]

Table S2. DNA concentration and purity determined by spectrophotometric analysis.

Buffer/column	DNA concentration [ng/µl]	260/280	260/230	Amount of DNA obtained [µg]
LB1/ SC1	35.55	2.03	2.28	5.33
LB1/ SC2	32.53	1.97	2.33	4.88
LB1/ SC3	25.25	2.18	2.94	3.79
LB1/ SCUN1	48.26	1.93	1.91	7.24
LB1/ SCUN2	45.18	1.91	1.97	6.78
LB1/ SC4	47.13	1.95	2	7.07
LB2/ SC1	36.93	1.91	2.23	5.54
LB2/ SC2	30.61	2.01	2.24	4.59
LB2/ SC3	33.82	2.08	2.58	5.07
LB2/ SCUN1	45.71	1.93	1.89	6.86
LB2/ SCUN2	49.13	1.92	1.87	7.37
LB2/ SC4	36.17	1.97	2.3	5.43
SB1/SC1	39.75	2.05	2.46	5.96
