# Supplementary figures and images for: A Method for Isolation Bacteriophage Particles-Free Genomic DNA, Exemplified by TP-84, Infecting Thermophilic Geobacillus
Source: Microorganisms. 2022 Sep 3;10(9):1782. doi: 10.3390/microorganisms10091782 (PMC9502220; doi:10.3390/microorganisms10091782)

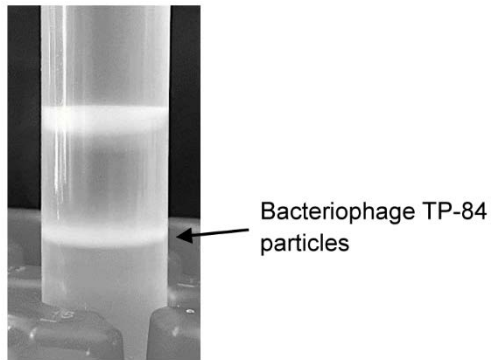

**Figure S1.** Bacteriophage TP-84 band after centrifugation in CsCl gradient.

Supplement: Supplementary file 1 [file microorganisms-10-01782-s001.zip › Figure S1.pdf]
